# Association of sleep quality and inner-ear–specific biomarkers Otolin-1 and otoconin-90 with disease severity in benign paroxysmal positional vertigo

**DOI:** 10.3389/fmed.2026.1769063

**Published:** 2026-02-27

**Authors:** Kui Li, Yuan-Hong He, Shuang-Jie Pan, Yuan-Zheng Zhao

**Affiliations:** Department of Neurology, The Fifth Affiliated Hospital of Zhengzhou University, Zhengzhou, Henan, China

**Keywords:** benign paroxysmal positional vertigo, Inner-ear biomarkers, otoconin-90, Otolin-1, sleep quality, vertigo severity

## Abstract

**Background:**

Benign paroxysmal positional vertigo (BPPV) is the most common peripheral vestibular disorder and exhibits marked heterogeneity in symptom burden and clinical course. Objective biomarkers reflecting inner-ear structural status and their relationship with clinical manifestations remain limited. Emerging evidence suggests an association between sleep quality and vertigo symptoms; however, the biological basis underlying this relationship is poorly understood.

**Methods:**

In this case–control study, 268 patients with BPPV and 268 age- and sex-matched healthy controls were enrolled. Sleep quality was assessed using the Pittsburgh Sleep Quality Index (PSQI), and vertigo severity was evaluated using the Dizziness Handicap Inventory (DHI). Serum levels of the inner-ear–specific structural biomarkers Otolin-1 and otoconin-90 (OC90) were measured using enzyme-linked immunosorbent assays. Logistic regression was used to examine associations with BPPV presence. Among patients with BPPV, multivariable linear regression, joint models, and exploratory mediation analyses were conducted to evaluate relationships among sleep quality, biomarkers, and symptom severity.

**Results:**

Compared with healthy controls, patients with BPPV exhibited significantly higher serum levels of the inner-ear structural biomarkers Otolin-1 (median 6.38 vs. 3.94 ng/mL) and otoconin-90 (median 12.64 vs. 7.58 ng/mL), both of which were independently associated with the odds of BPPV (adjusted OR for Otolin-1: 2.08, 95% CI: 1.49–2.91; adjusted OR for otoconin-90: 1.71, 95% CI: 1.21–2.42). Among patients with BPPV, poorer sleep quality was associated with greater vertigo severity (β = 2.14 per PSQI point, 95% CI: 1.56–2.72). Higher PSQI scores were also associated with increased levels of Otolin-1 and otoconin-90, both of which were independently related to vertigo severity. Inclusion of these biomarkers in joint models attenuated the PSQI–DHI association (β from 2.14 to 1.28) and improved model explanatory power (R^2^ from 0.26 to 0.38). Exploratory mediation analyses suggested that Otolin-1 and otoconin-90 statistically accounted for approximately 40 and 29% of the sleep–symptom association, respectively, with consistent findings across sensitivity and subgroup analyses.

**Conclusion:**

These findings indicate that otolith-related inner-ear structural biomarkers are associated with both the presence and severity of BPPV and may partially explain the relationship between sleep quality and vertigo symptoms.

## Introduction

Benign paroxysmal positional vertigo (BPPV) is one of the most common peripheral vestibular disorders and represents a substantial source of disability in clinical practice, with a pronounced impact on patients’ daily functioning and quality of life (1, 2). Although canalith repositioning maneuvers are generally effective, marked heterogeneity persists in clinical presentation and disease course (3, 4). From an epidemiological perspective, posterior semicircular canal BPPV represents the most prevalent subtype in the general and clinical populations, accounting for approximately 70–90% of cases in most series (5–7). Current clinical evaluation of BPPV relies largely on symptom-based scales and positional tests, which are essential for diagnosis but provide limited insight into the underlying biological state of the inner ear (8–10). As a result, clinicians have few objective tools to assess disease severity, explain inter-individual variability, or identify patients at risk of a more burdensome clinical course (4, 9, 11).

Biological indicators that reflect inner-ear structural status and correspond to symptom burden in BPPV remain limited (11–13). Otolin-1 is an extracellular matrix glycoprotein with expression largely restricted to the inner ear, where it contributes to the formation and stabilization of the organic otolith matrix, leading to its proposal as a potential biomarker reflecting otolith-related structural alterations (4, 11, 14). As a calcium-binding protein essential for otolith crystallization, Otoconin-90 (OC90) plays a critical role in maintaining otolith integrity and mineral organization (15, 16). Previous studies have suggested that abnormalities in OC90 expression or regulation may be linked to disrupted otolith metabolism, further supporting its relevance to otolith-associated vestibular disorders (15, 16). However, their clinical expression patterns and relevance in well-characterized patient populations remain insufficiently defined, underscoring the need for systematic evaluation in human studies (11, 15, 16).

An increasing body of evidence suggests that sleep quality is associated with vertigo-related symptoms and recurrence in patients with BPPV (17–19). However, most existing studies have approached this relationship primarily from a symptomatic perspective, focusing on subjective discomfort or functional impairment rather than underlying biological correlates (20, 21). Recent research has suggested that sleep disturbances in BPPV may not only be anxiety-derived but could also be the result of oxidative stress and other biological disruptions affecting inner-ear function (22–25). In this context, oxidative stress may exacerbate otolith damage, which in turn contributes to vertigo symptoms, further linking sleep disturbances to inner-ear structural alterations (23, 24, 26–28). However, it remains unclear whether variations in sleep quality are merely associated with symptom perception or reflect underlying alterations in inner-ear structural status that could partly account for the observed heterogeneity in clinical manifestations.

Despite growing interest in otolith-related mechanisms, important gaps remain in the current literature. Moreover, existing research has rarely integrated sleep quality, inner-ear structural biomarkers, and symptom severity within a unified analytical framework, limiting insight into how these factors may jointly contribute to the marked heterogeneity of clinical manifestations. To address these gaps, the present study aimed to comprehensively investigate the relationships among sleep quality, inner-ear–specific structural biomarkers (Otolin-1 and otoconin-90), and vertigo severity in a well-characterized cohort of patients with BPPV and clarify the clinical relevance of otolith-related biomarkers and to explore their potential role in linking sleep quality to symptom burden. Through this integrative analytical framework, we sought to provide clinical and biological insights into the heterogeneity of BPPV manifestations.

## Materials and methods

### Study design and study population

This study employed a case–control design, enrolling patients with BPPV as the case group and healthy individuals as the control group. A total of 268 consecutive patients with BPPV were recruited between January 2022 and June 2024 from the outpatient clinics, inpatient wards, and vertigo specialty center of The Fifth Affiliated Hospital of Zhengzhou University. All patients were evaluated by neurologists or otolaryngologists with expertise in vestibular disorders. Specifically, characteristic vertigo and positional nystagmus were elicited during the Dix–Hallpike maneuver or the supine roll test. Affected semicircular canals were classified as posterior canal, horizontal canal, or multiple canal involvement according to clinical and nystagmus features.

The control group consisted of 268 healthy volunteers recruited during the same period from the health examination center or community health screening programs. All control participants had no history of vertigo or balance disorders and no diagnosed vestibular or central nervous system diseases. Healthy controls were frequency-matched to patients with BPPV by age and sex, and comparability between groups in terms of body mass index (BMI) was assessed to minimize potential confounding.

This study was approved by the Medical Ethics Committee of The Fifth Affiliated Hospital of Zhengzhou University and was conducted in accordance with the principles of the Declaration of Helsinki. Written informed consent was obtained from all participants prior to enrollment.

### Inclusion and exclusion criteria

Patients were eligible for inclusion if they met all of the following criteria: (1) aged 18 years or older; (2) a confirmed diagnosis of BPPV according to the International Classification of Vestibular Disorders (ICVD) and relevant clinical guidelines, supported by typical clinical history and positive positional test findings; (3) ability to complete standardized questionnaires assessing sleep quality and vertigo severity; (4) willingness to provide peripheral blood samples for biomarker analysis; (5) provision of written informed consent.

Patients were excluded if any of the following conditions were present: (1) evidence of central causes of vertigo, including but not limited to cerebrovascular disease, intracranial tumors, or demyelinating disorders; (2) a history of major cerebrovascular events or other severe neurological diseases; (3) severe psychiatric or neurological disorders that could interfere with reliable assessment of sleep quality or vertigo-related symptoms; (4) severe hepatic or renal dysfunction, which might influence circulating protein levels; (5) use of medications within the preceding 4 weeks that could significantly affect vestibular function or sleep patterns, including vestibular suppressants, sedative-hypnotics, or psychoactive drugs; (6) incomplete clinical data or refusal to participate in questionnaire assessment or blood sampling.

### Clinical variables and questionnaire assessments

#### Baseline demographic and anthropometric characteristics

Baseline demographic and anthropometric data were collected for all participants at the time of enrollment. Age and sex were recorded based on self-report and medical records. Body mass index (BMI) was calculated as weight in kilograms divided by height in meters squared (kg/m^2^). Body weight and height were measured using standardized equipment during the clinical visit on the day of study inclusion.

#### Disease-related clinical characteristics

For patients with BPPV, disease-related clinical information was obtained through structured interviews and medical record review. Disease duration was defined as the time interval, in months, from the first documented episode of vertigo consistent with BPPV to the date of enrollment. Affected semicircular canal involvement was determined based on clinical presentation and nystagmus characteristics during positional testing and categorized as posterior canal, horizontal canal, or multiple canal involvement.

Recurrent BPPV was defined as the occurrence of one or more new episodes of positional vertigo consistent with BPPV following complete resolution of symptoms after appropriate canalith repositioning therapy, within the observation period prior to enrollment. This definition was applied consistently across all patients for descriptive and subgroup analyses.

#### Assessment of vertigo severity: Dizziness Handicap Inventory

Vertigo severity was assessed using the Dizziness Handicap Inventory (DHI), a validated self-report questionnaire designed to evaluate the impact of dizziness on daily functioning. The DHI consists of 25 items covering three domains: physical (P), emotional (E), and functional (F), with a total score ranging from 0 to 100, where higher scores indicate greater vertigo-related handicap. According to established criteria, DHI scores can be categorized as mild (0–30), moderate (31–60), or severe (61–100) impairment. In the present study, DHI was primarily analyzed as a continuous variable to preserve statistical power and capture the full spectrum of symptom severity. All participants completed the validated language version of the DHI at the time of enrollment, and the instrument has demonstrated good reliability in the study population.

### Assessment of sleep quality: Pittsburgh Sleep Quality Index

Sleep quality was evaluated using the PSQI, a widely used questionnaire that assesses sleep quality over the preceding month. The PSQI comprises 19 self-rated items, which are aggregated into seven component scores—subjective sleep quality, sleep latency, sleep duration, habitual sleep efficiency, sleep disturbances, use of sleep medication, and daytime dysfunction—and summed to yield a global score ranging from 0 to 21, with higher scores indicating poorer sleep quality. A global PSQI score greater than 5 is commonly used to define poor sleep quality.

### Measurement of inner-ear structural biomarkers

#### Blood collection and sample processing

Peripheral venous blood samples were collected from all participants on the day of enrollment, under standardized conditions. Blood was drawn in the morning after an overnight fast whenever feasible. Samples were allowed to clot at room temperature and were then centrifuged at 3,000 rpm for 10 min to separate serum. The resulting serum was aliquoted into pre-labeled tubes and stored at -80°C until analysis. To minimize degradation and variability, all samples were subjected to no more than one freeze–thaw cycle prior to biomarker measurement.

#### Biomarker assays

Serum concentrations of Otolin-1 and otoconin-90 (OC90) were quantified using commercially available enzyme-linked immunosorbent assay (ELISA) kits, according to the manufacturers’ instructions. For each assay, standard curves were generated using serial dilutions of recombinant standards. All samples were measured in duplicate, and the mean value was used for statistical analysis.

Assay performance was monitored by calculating intra-assay and inter-assay coefficients of variation (CVs), which were maintained within acceptable ranges specified by the manufacturers. Laboratory personnel performing the assays were blinded to clinical information, and samples were analyzed in a randomized order to reduce potential batch effects. Quality control samples were included across assay plates to ensure consistency and reliability of measurements.

#### Units and data preprocessing

Serum concentrations of Otolin-1 and OC90 were expressed in nanograms per milliliter (ng/mL). Distributional assessment revealed that both biomarkers exhibited right-skewed distributions. Therefore, logarithmic transformation was applied prior to regression analyses to improve normality and stabilize variance.

### Statistical analysis

All statistical analyses were performed using R software and SPSS Statistics. All tests were two-sided, and a *P* < 0.05 was considered statistically significant. *P*-values were reported to three decimal places, consistent with the presentation of results in tables and figures. The extent of missing data was assessed for all variables prior to analysis. As the proportion of missing values was minimal, analyses were conducted using a complete-case approach. Participants with missing key clinical variables, questionnaire data, or biomarker measurements were excluded from the corresponding analyses.

Normality of continuous variables was evaluated using the Shapiro–Wilk test and visual inspection of distributions. Continuous variables with approximately normal distributions were summarized as mean ± standard deviation (SD), whereas non-normally distributed variables were presented as median (interquartile range, IQR). Categorical variables were expressed as counts and percentages. Between-group comparisons were performed using the independent-samples *t*-test for normally distributed continuous variables and the Mann–Whitney U test for non-normally distributed variables. Categorical variables were compared using the χ^2^ test or Fisher’s exact test, as appropriate.

Logistic regression models were used to evaluate the associations between inner-ear structural biomarkers and the presence of BPPV. BPPV status was treated as the dependent variable (1 = BPPV, 0 = healthy control). Independent variables included log-transformed serum Otolin-1 and log-transformed otoconin-90 (OC90), which were analyzed in separate models. Two models were constructed: Model 1, an unadjusted model, and Model 2, adjusted for age, sex, and body mass index (BMI). Results were reported as odds ratios (ORs) with 95% confidence intervals (CIs) and corresponding *P*-values.

Among patients with BPPV, linear regression analyses were conducted to examine the association between sleep quality and vertigo severity. The Dizziness Handicap Inventory (DHI) total score was modeled as a continuous dependent variable, with the PSQI global score as the primary independent variable. Models were adjusted for age, sex, BMI, disease duration, and affected semicircular canal type. Results were expressed as regression coefficients (β) with 95% CIs and *P*-values. Correlation analyses using Pearson’s or Spearman’s correlation coefficients, as appropriate, were performed as Supplementary analyses.

Linear regression models were used to assess the associations between sleep quality and inner-ear structural biomarkers (Table 3). Log-transformed serum Otolin-1 and OC90 levels were analyzed as dependent variables, with the PSQI global score as the independent variable. Unadjusted models (Model 1) and multivariable-adjusted models (Model 2) were fitted. Model 2 included adjustment for age, sex, BMI, and relevant clinical characteristics. Regression coefficients (β), 95% CIs, and *P*-values were reported.

To examine the relationships between inner-ear biomarkers and vertigo severity, linear regression models were constructed with DHI total score as the dependent variable. Otolin-1 and OC90 were first analyzed in separate models, followed by a joint model including both biomarkers simultaneously. All models were adjusted for the same covariates as the primary analyses. Multicollinearity was assessed using the variance inflation factor (VIF), with values < 5 indicating no substantial collinearity.

Nested multivariable linear regression models were constructed to evaluate the combined contributions of sleep quality and inner-ear biomarkers to vertigo severity. Four models were specified: Model A included PSQI only; Model B included PSQI and Otolin-1; Model C included PSQI and OC90; and Model D included PSQI, Otolin-1, and OC90 simultaneously. All models were adjusted for the same set of covariates. Changes in regression coefficients for PSQI and model explanatory power (R^2^) were examined to assess the extent to which biomarker inclusion attenuated the association between sleep quality and vertigo severity.

Exploratory mediation analyses were conducted to examine whether inner-ear structural biomarkers statistically account for part of the association between sleep quality and vertigo severity. Sleep quality (PSQI score) was specified as the exposure, log-transformed Otolin-1 or OC90 as the mediator, and DHI total score as the outcome. Covariates included age, sex, BMI, and the same clinical variables used in the primary regression models. Mediation effects were estimated using regression-based mediation analysis with bootstrap resampling (5,000 iterations) to derive 95% confidence intervals for indirect effects. Total, direct, and indirect effects were calculated, along with the proportion mediated. These analyses were exploratory in nature and intended to provide statistical interpretation rather than causal inference.

Sensitivity analyses were performed to evaluate the robustness of the primary findings. First, analyses were repeated after excluding patients with recurrent BPPV, restricting the sample to first-episode cases. Second, subgroup analyses were conducted according to affected semicircular canal type, comparing posterior canal BPPV with non-posterior canal involvement. Key regression models were re-estimated within each subgroup to assess consistency of associations.

## Results

### Baseline characteristics of the study population

A total of 268 patients with benign paroxysmal positional vertigo were included in the analysis (Table 1). The mean age of the study population was 53.47 ± 11.26 years, and 190 patients (70.90%) were female. The mean body mass index was 22.84 ± 3.12 kg/m^2^. The median disease duration was 3.60 months (interquartile range, 1.20–9.80 months). The two groups were comparable in terms of age, sex distribution, and body mass index, with no statistically significant differences observed. Patients with BPPV exhibited significantly poorer sleep quality, as reflected by higher PSQI scores compared with healthy controls.

**TABLE 1:** Baseline characteristics of patients with benign paroxysmal positional vertigo and healthy controls.

Characteristic	BPPV (*n* = 268)	Healthy controls (*n* = 268)	*P-*value
Age, years	53.47 ± 11.26	52.88 ± 10.94	0.534
Sex, n (%)			0.447
Male	78 (29.10)	84 (31.34)	
Female	190 (70.90)	184 (68.66)	
Body mass index, kg/m^2^	22.84 ± 3.12	23.06 ± 3.25	0.412
Disease duration, months	3.60 (1.20–9.80)	/	—
Affected semicircular canal, n (%)		/	—
Posterior canal	189 (70.52)	/	
Horizontal canal	59 (22.01)	/	
Multiple canals	20 (7.46)	/	
Recurrent BPPV, n (%)	92 (34.33)	/	—
Sleep quality (PSQI score)	8.42 ± 3.11	5.36 ± 2.42	< 0.001
Vertigo severity (DHI score)	46.75 ± 18.92	/	—
Serum Otolin-1, ng/mL	6.38 (4.92–8.71)	3.94 (3.12–5.06)	< 0.001
Serum otoconin-90, ng/mL	12.64 (9.35–17.18)	7.58 (5.94–9.66)	< 0.001

Values are presented as mean ± standard deviation for normally distributed continuous variables, median (interquartile range) for non-normally distributed variables, and number (percentage) for categorical variables. Comparisons between patients with BPPV and healthy controls were performed using the independent-samples *t*-test or Mann–Whitney *U* test for continuous variables, as appropriate, and the χ^2^-test for categorical variables. *P*-values are reported to three decimal places. PSQI indicates Pittsburgh Sleep Quality Index; DHI, Dizziness Handicap Inventory; BPPV, benign paroxysmal positional vertigo.

Regarding the affected semicircular canals, posterior canal involvement was the most common presentation, observed in 189 patients (70.52%), followed by horizontal canal involvement in 59 patients (22.01%), and multiple canal involvement in 20 patients (7.46%). Recurrent BPPV was identified in 92 patients (34.33%).

The mean Pittsburgh Sleep Quality Index (PSQI) score was 8.42 ± 3.11, indicating a high prevalence of impaired sleep quality in the cohort. The mean Dizziness Handicap Inventory (DHI) score was 46.75 ± 18.92, reflecting a moderate overall burden of vertigo-related disability. Median serum levels of Otolin-1 and Otoconin-90 were 6.38 ng/mL (interquartile range, 4.92–8.71 ng/mL) and 12.64 ng/mL (interquartile range, 9.35–17.18 ng/mL), respectively. Serum levels of the inner-ear–specific biomarkers Otolin-1 and Otoconin-90 were both significantly higher in patients with BPPV than in controls (Table 1). The distribution of the key study variables is shown in Supplementary Figure 1.

### Associations of inner-ear structural biomarkers with the odds of BPPV

As shown in Table 2, higher serum levels of both inner-ear structural biomarkers were significantly associated with the presence of benign paroxysmal positional vertigo. In unadjusted logistic regression models, each one-unit increase in log-transformed serum Otolin-1 was associated with a 2.41-fold increase in the odds of BPPV (OR = 2.41, 95% CI: 1.78–3.26, *P* < 0.001), while Otoconin-90 was associated with a 1.96-fold increase in the odds of BPPV (OR = 1.96, 95% CI: 1.44–2.68, *P* < 0.001).

**TABLE 2:** Associations of inner-ear structural biomarkers with the odds of benign paroxysmal positional vertigo.

Variable	Model 1 OR (95% CI)	*P-*value	Model 2 OR (95% CI)	*P*-value
Serum Otolin-1 (log-transformed)	2.41 (1.78–3.26)	< 0.001	2.08 (1.49–2.91)	< 0.001
Serum otoconin-90 (log-transformed)	1.96 (1.44–2.68)	< 0.001	1.71 (1.21–2.42)	0.002

Logistic regression analyses were performed with BPPV status (yes/no) as the dependent variable. Model 1 represents the unadjusted model. Model 2 was adjusted for age, sex, and body mass index. Serum Otolin-1 and Otoconin-90 levels were log-transformed prior to analysis due to right-skewed distributions. Results are presented as odds ratios (ORs) with 95% confidence intervals (CI). All statistical tests were two-sided, and *P* values are reported to three decimal places. BPPV indicates benign paroxysmal positional vertigo.

After adjustment for age, sex, and body mass index, these associations remained statistically significant. Specifically, the adjusted odds ratio for Otolin-1 was 2.08 (95% CI: 1.49–2.91, *P* < 0.001), whereas Otoconin-90 showed an adjusted odds ratio of 1.71 (95% CI: 1.21–2.42, *P* = 0.002).

### Association between sleep quality and vertigo severity

As shown in Table 3, poorer sleep quality was significantly associated with greater vertigo severity. In linear regression analyses, each 1-point increase in PSQI score was associated with a 2.38-point increase in total DHI score in the unadjusted model (β = 2.38, 95% CI: 1.81–2.95; *P* < 0.001). This association remained statistically significant after adjustment for age, sex, body mass index, disease duration, and affected semicircular canal type (β = 2.14, 95% CI: 1.56–2.72; *P* < 0.001), indicating a robust relationship between sleep quality and vertigo-related disability. Exploratory analyses of individual PSQI component scores in relation to vertigo severity are presented in Supplementary Table 1.

**TABLE 3:** Association between sleep quality (PSQI) and Vertigo severity (DHI).

Model	Exposure variable	Outcome variable	β (95% confidence interval)	*P*-value
Unadjusted	PSQI score (per 1-point increase)	Total DHI score	2.38 (1.81–2.95)	< 0.001
Adjusted	PSQI score (per 1-point increase)	Total DHI score	2.14 (1.56–2.72)	< 0.001

Regression coefficients are presented as β values with 95% confidence intervals (CI), indicating the estimated change in DHI score associated with each 1-point increase in PSQI score. The adjusted model was controlled for age, sex, body mass index, disease duration, and affected semicircular canal type. All statistical tests were two-sided. P values are reported to three decimal places. PSQI indicates Pittsburgh Sleep Quality Index; DHI, Dizziness Handicap Inventory.

### Association between sleep quality and inner-ear structural biomarkers

As shown in Table 4, poorer sleep quality was significantly associated with higher levels of inner-ear structural biomarkers. In unadjusted linear regression analyses, higher PSQI scores were associated with increased serum Otolin-1 levels (β = 0.08, 95% CI: 0.05–0.11; *P* < 0.001) and Otoconin-90 levels (β = 0.06, 95% CI: 0.03–0.09; *P* < 0.001). These associations remained statistically significant after adjustment for age, sex, body mass index, disease duration, and affected semicircular canal type. In adjusted models, the magnitude of association was slightly attenuated but remained robust for both Otolin-1 (β = 0.07, 95% CI: 0.04–0.10; *P* < 0.001) and Otoconin-90 (β = 0.05, 95% CI: 0.02–0.08; *P* = 0.001).

**TABLE 4:** Association between sleep quality and inner-ear structural biomarkers.

Outcome variable	Model	Exposure variable	β (95% confidence interval)	*P*-value
Serum Otolin-1 (log-transformed)	Unadjusted	PSQI score (per 1-point increase)	0.08 (0.05–0.11)	< 0.001
	Adjusted	PSQI score (per 1-point increase)	0.07 (0.04 to 0.10)	< 0.001
Serum otoconin-90 (log-transformed)	Unadjusted	PSQI score (per 1-point increase)	0.06 (0.03–0.09)	< 0.001
	Adjusted	PSQI score (per 1-point increase)	0.05 (0.02–0.08)	0.001

Regression coefficients are presented as β values with 95% confidence intervals (CI), representing the estimated change in log-transformed biomarker levels per 1-point increase in PSQI score. The adjusted model was controlled for age, sex, body mass index, disease duration, and affected semicircular canal type. All statistical tests were two-sided. *P* values are reported to three decimal places. PSQI indicates Pittsburgh Sleep Quality Index.

### Association between otolin-1, OC90, and vertigo severity

As presented in Table 5, higher levels of inner-ear structural biomarkers were significantly associated with greater vertigo severity. In unadjusted linear regression analyses, both serum Otolin-1 and Otoconin-90 levels were positively associated with total DHI score (Otolin-1: β = 9.42, 95% CI: 6.21–12.63; *P* < 0.001; OC90: β = 7.15, 95% CI: 4.02–10.28; *P* < 0.001). These associations remained statistically significant after adjustment for age, sex, body mass index, disease duration, and affected semicircular canal type.

**TABLE 5:** Associations of inner-ear structural biomarkers with vertigo severity.

Model	Exposure variable	Outcome variable	β (95% confidence interval)	*P*-value
Unadjusted	Serum Otolin-1 (log-transformed)	Total DHI score	9.42 (6.21–12.63)	< 0.001
Unadjusted	Serum otoconin-90 (log-transformed)	Total DHI score	7.15 (4.02–10.28)	< 0.001
Adjusted	Serum Otolin-1 (log-transformed)	Total DHI score	8.36 (5.14–11.58)	< 0.001
Adjusted	Serum otoconin-90 (log-transformed)	Total DHI score	6.02 (2.91–9.13)	< 0.001
Joint model	Serum Otolin-1 (log-transformed)	Total DHI score	6.84 (3.58–10.10)	< 0.001
Joint model	Serum otoconin-90 (log-transformed)	Total DHI score	4.21 (1.18–7.24)	0.007

Multiple linear regression analyses were performed with Dizziness Handicap Inventory (DHI) total score as a continuous dependent variable. Regression coefficients are presented as β values with 95% confidence intervals (CI), representing the estimated change in DHI score associated with a one-unit increase in log-transformed biomarker levels. Adjusted and joint models were controlled for age, sex, body mass index, disease duration, and affected semicircular canal type. All statistical tests were two-sided. *P*-values are reported to three decimal places. DHI indicates Dizziness Handicap Inventory.

When both biomarkers were included simultaneously in the joint model, Otolin-1 and Otoconin-90 remained independently associated with vertigo severity, although the magnitude of the associations was moderately attenuated (Otolin-1: β = 6.84, 95% CI: 3.58–10.10; *P* < 0.001; OC90: β = 4.21, 95% CI: 1.18–7.24; *P* = 0.007).

### Joint models including sleep quality and inner-ear biomarkers

Results of the joint regression models are summarized in Table 6. In Model A, poorer sleep quality was significantly associated with greater vertigo severity, with each 1-point increase in PSQI score corresponding to a 2.14-point increase in total DHI score (β = 2.14, 95% CI: 1.56–2.72; *P* < 0.001).

**TABLE 6:** Joint models of sleep quality and inner-ear biomarkers for vertigo severity.

Model	Predictors	β (95% confidence interval)	*P-*value	*R* ^2^
Model A	PSQI score (per 1-point increase)	2.14 (1.56–2.72)	< 0.001	0.26
Model B	PSQI score	1.62 (1.05–2.19)	< 0.001	0.33
	Otolin-1 (log-transformed)	6.11 (3.12–9.10)	< 0.001	
Model C	PSQI score	1.74 (1.17–2.31)	< 0.001	0.31
	Otoconin-90 (log-transformed)	4.02 (1.08–6.96)	0.007	
Model D	PSQI score	1.28 (0.74–1.82)	< 0.001	0.38
	Otolin-1 (log-transformed)	4.58 (1.62–7.54)	0.002	
	Otoconin-90 (log-transformed)	2.93 (0.24–5.62)	0.033	

Multiple linear regression analyses were conducted with Dizziness Handicap Inventory (DHI) total score as a continuous dependent variable. Model A included sleep quality only, with the Pittsburgh Sleep Quality Index (PSQI) score entered as the primary independent variable. Model B included PSQI score and serum Otolin-1 (log-transformed). Model C included PSQI score and serum Otoconin-90 (log-transformed). Model D included PSQI score, serum Otolin-1, and serum Otoconin-90 simultaneously (both biomarkers log-transformed). All models were adjusted for age, sex, body mass index, disease duration, and affected semicircular canal type. Regression coefficients are presented as β values with 95% confidence intervals (CI), representing the estimated change in DHI score per unit increase in each predictor. Model performance was evaluated using the coefficient of determination (R^2^). All statistical tests were two-sided, and *P*-values are reported to three decimal places. PSQI indicates Pittsburgh Sleep Quality Index; DHI, Dizziness Handicap Inventory.

After inclusion of Otolin-1 in Model B, the association between PSQI and DHI was attenuated but remained statistically significant (β = 1.62, 95% CI: 1.05–2.19; *P* < 0.001), while Otolin-1 showed an independent positive association with vertigo severity. A similar pattern was observed in Model C, in which adjustment for Otoconin-90 resulted in a modest reduction in the PSQI effect (β = 1.74, 95% CI: 1.17–2.31; *P* < 0.001), and Otoconin-90 remained significantly associated with DHI.

In the fully adjusted Model D, which included PSQI, Otolin-1, and Otoconin-90 simultaneously, all three variables remained independently associated with vertigo severity. Compared with Model A, the regression coefficient for PSQI was further reduced (β = 1.28, 95% CI: 0.74–1.82; *P* < 0.001), whereas both Otolin-1 and Otoconin-90 retained statistically significant associations. The explanatory power of the model increased progressively with the addition of inner-ear biomarkers, with R^2^ rising from 0.26 in Model A to 0.38 in Model D.

### Exploratory mediation analysis of inner-ear structural biomarkers

Exploratory mediation analyses were conducted to examine whether inner-ear–specific structural biomarkers statistically accounted for part of the association between sleep quality and vertigo severity (Table 7). When Otolin-1 was evaluated as a mediator, the indirect effect was statistically significant (effect estimate = 0.86, 95% CI: 0.42–1.30; *P* < 0.001), accounting for approximately 40.2% of the total association between PSQI score and DHI score. Similarly, analyses including Otoconin-90 as a mediator demonstrated a significant indirect effect (effect estimate = 0.62, 95% CI: 0.28–0.96; *P* = 0.001), corresponding to a mediation proportion of approximately 29.0%.

**TABLE 7:** Exploratory mediation analysis of inner-ear structural biomarkers in the association between sleep quality and vertigo severity.

Mediator	Effect type	Effect estimate	95% confidence interval	*P*-value	Proportion mediated (%)
Otolin-1	Total effect	2.14	1.56–2.72	< 0.001	—
	Direct effect	1.28	0.74–1.82	<0.001	—
	Indirect effect	0.86	0.42–1.30	< 0.001	40.19
Otoconin-90	Total effect	2.14	1.56–2.72	< 0.001	—
	Direct effect	1.52	0.96–2.08	<0.001	—
	Indirect effect	0.62	0.28–0.96	0.001	28.97

All models were adjusted for age, sex, body mass index, disease duration, and affected semicircular canal type, consistent with the main regression analyses. Effect estimates represent changes in DHI score per 1-point increase in PSQI. The proportion mediated was calculated as the ratio of the indirect effect to the total effect and is reported as a percentage. All statistical tests were two-sided. *P*-values are reported to three decimal places. PSQI indicates Pittsburgh Sleep Quality Index; DHI, Dizziness Handicap Inventory.

### Sensitivity and subgroup analyses

Results of sensitivity and subgroup analyses are presented in Supplementary Tables 2–3. After excluding patients with recurrent BPPV, the associations between sleep quality, inner-ear structural biomarkers, and vertigo severity remained directionally consistent with the primary analyses, with comparable effect estimates observed for PSQI, Otolin-1, and Otoconin-90 (Supplementary Table 2).

Subgroup analyses stratified by affected semicircular canal type demonstrated similar associations across posterior and non-posterior canal involvement, with no evidence that a specific canal subtype disproportionately influenced the results (Supplementary Table 3). Likewise, analyses stratified by sex showed consistent associations in both male and female participants (Supplementary Table 3).

## Discussion

In this study, we employed a case–control design to examine the relationships among sleep quality, Otolin-1 and otoconin-90, and clinical manifestations of BPPV. Several key findings emerged. First, serum levels of Otolin-1 and otoconin-90 were elevated in patients with BPPV and were associated with disease presence. Second, poorer sleep quality was associated with greater vertigo-related symptom burden among patients with BPPV. Third, sleep quality was also related to levels of otolith-associated structural biomarkers, and the strength of the sleep–symptom association was attenuated after accounting for these biomarkers, accompanied by improved model explanatory power.

Otolin-1 and otoconin-90 are inner-ear–specific matrix proteins that play important roles in otolith formation and structural stability (16, 29, 30). In the present study, both biomarkers were found to be elevated in patients with BPPV compared with healthy controls and were independently associated with the odds of BPPV. These findings are consistent with prevailing pathophysiological concepts suggesting that otolith detachment or altered otolith metabolism underlies the development of BPPV (4, 11, 12, 14, 16, 30–33). Notably, Otolin-1 demonstrated a slightly stronger association with disease presence than otoconin-90, which may indicate greater sensitivity to structural alterations of the otolith matrix. By demonstrating these associations in a well-characterized clinical population, our results extend existing experimental and mechanistic evidence and support the potential clinical relevance of otolith-related biomarkers in BPPV.

In this study, poorer sleep quality was significantly associated with greater vertigo-related symptom severity among patients with BPPV, as reflected by higher DHI scores. This finding indicates that sleep status is closely related to the subjective symptom burden experienced by patients. Such an association is in line with previous observations that sleep disturbances frequently coexist with vestibular symptoms (17, 19, 21, 34–38). However, most prior studies have focused primarily on symptomatic correlations, with limited exploration of the underlying biological context (20, 21, 39, 40). By establishing a robust association between sleep quality and vertigo severity, the present findings provide an essential clinical foundation for subsequent analyses examining how sleep may relate to inner-ear structural alterations and symptom heterogeneity.

Beyond its association with symptom severity, this study further demonstrated that sleep quality was significantly related to serum levels of the otolith-associated structural biomarkers Otolin-1 and otoconin-90. This observation suggests that sleep status may be linked to otolith-related structural alterations, indicating that these factors could operate within a shared biological framework rather than representing isolated clinical features (41–46). Although the underlying mechanisms remain unclear, such associations may involve changes in inner-ear microenvironmental homeostasis, metabolic balance, or clearance processes that influence otolith integrity (44, 47). By identifying a relationship between sleep quality and inner-ear structural biomarkers, these findings extend the traditional sleep–vertigo association toward a structural perspective of inner-ear status.

In addition to their associations with disease presence, both Otolin-1 and otoconin-90 were independently associated with vertigo severity among patients with BPPV. When examined in separate models, higher levels of each biomarker were related to greater DHI scores. Importantly, these associations remained statistically significant in joint models including both biomarkers, although partial attenuation of effect estimates was observed. This pattern suggests that Otolin-1 and otoconin-90 capture overlapping yet non-identical aspects of otolith-related structural status. Together, these findings support the notion that variations in otolith structure may independently contribute to differences in symptom burden and further strengthen the rationale for considering these molecules as clinically relevant biological indicators in BPPV.

In the joint models, the association between sleep quality and vertigo severity was notably attenuated after the inclusion of Otolin-1 and otoconin-90, accompanied by a meaningful improvement in model explanatory power. This pattern indicates that otolith-related structural biomarkers statistically account for part of the observed relationship between sleep quality and symptom burden. Consistent with this observation, exploratory mediation analyses further suggested that these biomarkers explain a proportion of the sleep–symptom association at a statistical level. Importantly, these findings do not support a single-pathway interpretation but are more consistent with a multifactorial framework in which partially overlapping biological pathways jointly contribute to clinical heterogeneity. From a methodological perspective, the convergence of results across joint regression and mediation analyses strengthens the internal coherence and credibility of the study findings.

The robustness of the primary findings was supported by a series of sensitivity and subgroup analyses. After excluding patients with recurrent BPPV, the main associations among sleep quality, inner-ear structural biomarkers, and vertigo severity remained directionally consistent. Similar patterns were observed across subgroups stratified by affected semicircular canal type and sex. These results indicate that the observed associations are unlikely to be driven by a specific clinical subtype or demographic characteristic and enhance the generalizability of the findings across different BPPV subpopulations.

Clinically, our results support a more integrative view of BPPV in which symptom burden may be influenced by underlying otolith-related biological alterations in addition to perceptual and psychological factors. While immediate clinical application is premature, these findings may assist clinicians in interpreting symptom heterogeneity and highlight sleep quality as a potentially relevant, modifiable factor in patient assessment.

At present, our study has focused exclusively on BPPV; however, the biological roles of Otolin-1 and Otoconin-90 suggest that these biomarkers may also be relevant in other vestibular disorders associated with otolith dysfunction, such as Meniere’s disease, vestibular neuritis, or bilateral vestibular hypofunction. Future studies will be essential to explore the potential diagnostic and prognostic value of these biomarkers in a broader range of vertigo-related conditions.

Several limitations should be acknowledged. First, the cross-sectional nature of this study precludes establishing temporal ordering and does not allow causal inference regarding the observed associations among sleep quality, inner-ear biomarkers, and symptom severity. Second, Otolin-1 and otoconin-90 were measured at a single time point, and thus may not capture within-individual variability or dynamic changes across disease episodes or treatment responses. Third, the control group consisted of healthy individuals; the absence of disease controls with other causes of dizziness limits the specificity assessment of these biomarkers for BPPV. Fourth, although key demographic and clinical covariates were considered, residual confounding remains possible because factors such as vitamin D status, bone mineral density, and medication exposures were not comprehensively assessed. Based on their biological roles as otolith-related structural proteins, it is reasonable to hypothesize that Otolin-1 and Otoconin-90 concentrations may decrease after successful canalith repositioning, with potential changes occurring over a short-to-intermediate time course rather than immediately, and possibly lagging behind subjective symptom resolution. Finally, the mediation analyses were exploratory and intended to provide statistical interpretation; their results should be interpreted cautiously and warrant validation in well-designed prospective and longitudinal studies.

## Conclusion

In conclusion, this study demonstrates that the inner-ear structural biomarkers Otolin-1 and otoconin-90 are elevated in patients with benign paroxysmal positional vertigo and are associated with both disease presence and vertigo severity. Sleep quality was significantly related to symptom burden and was also associated with levels of otolith-related structural biomarkers. Joint analyses further suggest that these biomarkers statistically account for part of the association between sleep quality and vertigo severity. Taken together, these findings provide new insights into the clinical heterogeneity of BPPV from a structural biological perspective and lay a foundation for future prospective and mechanistic studies.

## Data Availability

The original contributions presented in the study are included in the article/Supplementary material, further inquiries can be directed to the corresponding author.
