# Supplementary material for: Association of sleep quality and inner-ear–specific biomarkers Otolin-1 and otoconin-90 with disease severity in benign paroxysmal positional vertigo
Source: Front Med (Lausanne). 2026 Feb 27;13:1769063. doi: 10.3389/fmed.2026.1769063 (PMC12982452; doi:10.3389/fmed.2026.1769063)
Supplement: Supplementary file 1 [file Data_Sheet_1.pdf]

```
# BPPV analysis pipeline (Python)
# -----------------------------------------------------------
# Purpose:
#   Reproduce primary analyses reported in the manuscript:
#   - Descriptive statistics (Table 1)
#   - Distribution plots (Figure S1)
#   - Logistic regression for BPPV presence (Table 6)
#   - Linear regression models within BPPV cases (Tables 2–5)
#   - Joint nested models (Table 5 in manuscript; adjust numbering as needed)
#   - Exploratory mediation (bootstrapped) (Table 7 / Supplementary)
#   - Sensitivity / subgroup analyses (Supplementary)
#
# Inputs:
#   An Excel file with at least these columns:
#     Group (BPPV/Control), Age, Sex, BMI, PSQI,
#     Otolin1_ng_ml, OC90_ng_ml,
#   and (BPPV-only, optional but recommended):
#     DHI, Disease_duration_months, Canal_type, Recurrent (0/1)
#
# Output:
#   Model summaries printed to console and optionally exported to CSV.
#
# NOTE:
#   This script is designed to run on your real dataset.
#   Replace FILE_PATH and verify column names match your sheet.

import numpy as np
import pandas as pd
from scipy import stats
import statsmodels.api as sm
import statsmodels.formula.api as smf
import matplotlib.pyplot as plt

FILE_PATH = "BPPV_raw_simulated_dataset.xlsx"  # <- replace with your file
OUTDIR = "analysis_outputs"
os.makedirs(OUTDIR, exist_ok=True)

# -----------------------
# 1) Load and preprocess
# -----------------------
df = pd.read_excel(FILE_PATH)

# Normalize group labels
df["Group"] = df["Group"].astype(str).str.strip()
df["is_bppv"] = (df["Group"].str.lower() == "bppv").astype(int)

# Encode sex
df["Sex"] = df["Sex"].astype(str).str.strip()
df["Sex_male"] = (df["Sex"].str.lower() == "male").astype(int)

# Log-transform biomarkers (right-skewed)
df["log_Otolin1"] = np.log(df["Otolin1_ng_ml"])
df["log_OC90"] = np.log(df["OC90_ng_ml"])

# Split groups
cases = df[df["is_bppv"] == 1].copy()
ctrls = df[df["is_bppv"] == 0].copy()

# -----------------------
# 2) Helper functions
# -----------------------
def shapiro_p(x):
    x = pd.Series(x).dropna()
    if len(x) < 3:
        return np.nan
    return stats.shapiro(x.sample(min(len(x), 5000), random_state=1))[1]

def describe_continuous(x):
```

```
    x = pd.Series(x).dropna()
    if len(x) == 0:
        return {"n": 0}
    p = shapiro_p(x)
    if p is not np.nan and p >= 0.05:
        return {"n": len(x), "type": "mean_sd", "mean": x.mean(), "sd": x.std(ddof=1), "p_norm": p}
    else:
        return {"n": len(x), "type": "median_iqr", "median": x.median(),
                "q1": x.quantile(0.25), "q3": x.quantile(0.75), "p_norm": p}

def pvalue_group_compare(x_case, x_ctrl):
    # Normality-based choice: t test vs Mann-Whitney
    x_case = pd.Series(x_case).dropna()
    x_ctrl = pd.Series(x_ctrl).dropna()
    if len(x_case) < 3 or len(x_ctrl) < 3:
        return np.nan, "NA"
    p1, p2 = shapiro_p(x_case), shapiro_p(x_ctrl)
    if (p1 is not np.nan and p1 >= 0.05) and (p2 is not np.nan and p2 >= 0.05):
        p = stats.ttest_ind(x_case, x_ctrl, equal_var=False).pvalue
        return p, "t-test"
    p = stats.mannwhitneyu(x_case, x_ctrl, alternative="two-sided").pvalue
    return p, "Mann-Whitney"

# -----------------------
# 3) Table 1 style summary
# -----------------------
table1_vars = ["Age", "BMI", "PSQI", "Otolin1_ng_ml", "OC90_ng_ml"]
table1 = []
for v in table1_vars:
    desc_cases = describe_continuous(cases[v])
    desc_ctrls = describe_continuous(ctrls[v])
    p, test = pvalue_group_compare(cases[v], ctrls[v])
    table1.append({
        "Variable": v,
        "Cases_summary": desc_cases,
        "Controls_summary": desc_ctrls,
        "P_value": p,
        "Test": test
    })
table1_df = pd.DataFrame(table1)
table1_df.to_csv(os.path.join(OUTDIR, "Table1_descriptives.csv"), index=False)

# Categorical example: Sex
sex_tab = pd.crosstab(df["Group"], df["Sex"])
chi2_p = stats.chi2_contingency(sex_tab)[1] if sex_tab.size > 0 else np.nan

# -----------------------
# 4) Figure S1 (distributions)
# -----------------------
for biom, logcol in [("Otolin1_ng_ml", "log_Otolin1"), ("OC90_ng_ml", "log_OC90")]:
    x = cases[biom].dropna()
    plt.figure()
    plt.hist(x, bins=25, density=True)
    x_grid = np.linspace(x.min(), x.max(), 200)
    # kernel density (simple Gaussian KDE)
    kde = stats.gaussian_kde(x)
    plt.plot(x_grid, kde(x_grid))
    plt.title(f"{biom}: histogram + density (BPPV)")
    plt.xlabel(biom)
    plt.ylabel("Density")
    plt.tight_layout()
    plt.savefig(os.path.join(OUTDIR, f"Figure_S1_{biom}.png"), dpi=300)
    plt.close()

# -----------------------
# 5) Logistic regression (Table 6)
#    Outcome: BPPV (yes/no)
# -----------------------
```

```
# Model 1: unadjusted
m1_otolin = smf.logit("is_bppv ~ log_Otolin1", data=df).fit(disp=0)
m1_oc90   = smf.logit("is_bppv ~ log_OC90", data=df).fit(disp=0)

# Model 2: adjusted
m2_otolin = smf.logit("is_bppv ~ log_Otolin1 + Age + Sex_male + BMI", data=df).fit(disp=0)
m2_oc90   = smf.logit("is_bppv ~ log_OC90 + Age + Sex_male + BMI", data=df).fit(disp=0)

def or_ci(model, term):
    b = model.params[term]
    se = model.bse[term]
    lo, hi = b - 1.96*se, b + 1.96*se
    return np.exp(b), np.exp(lo), np.exp(hi), model.pvalues[term]

logit_rows = []
for name, model, term in [
    ("Otolin-1", m1_otolin, "log_Otolin1"),
    ("OC90",     m1_oc90,   "log_OC90"),
    ("Otolin-1", m2_otolin, "log_Otolin1"),
    ("OC90",     m2_oc90,   "log_OC90"),
]:
    OR, L, U, p = or_ci(model, term)
    logit_rows.append({"Biomarker": name, "Model": "Unadjusted" if "m1" in model.model.formula else
"Adjusted",
                       "OR": OR, "CI_low": L, "CI_high": U, "P": p})
pd.DataFrame(logit_rows).to_csv(os.path.join(OUTDIR, "Table6_logistic.csv"), index=False)

# -----------------------
# 6) Linear models in BPPV cases
# -----------------------
# Table 2: DHI ~ PSQI (adjusted)
# NOTE: Requires DHI not missing for cases
if "DHI" in cases.columns and cases["DHI"].notna().any():
    # Core covariates; add disease variables if present
    covs = ["Age", "Sex_male", "BMI"]
    if "Disease_duration_months" in cases.columns and
cases["Disease_duration_months"].notna().any():
        covs.append("Disease_duration_months")
    if "Canal_type" in cases.columns:
        # Use categorical if present; statsmodels will handle C()
        formula_adj = "DHI ~ PSQI + " + " + ".join([c if c != "Canal_type" else "C(Canal_type)" for
c in covs])
    else:
        formula_adj = "DHI ~ PSQI + " + " + ".join(covs)

    lm_psqi_unadj = smf.ols("DHI ~ PSQI", data=cases).fit()
    lm_psqi_adj   = smf.ols(formula_adj, data=cases).fit()

    # Table 3: log biomarker ~ PSQI (adjusted)
    lm_otolin_psqi = smf.ols("log_Otolin1 ~ PSQI + Age + Sex_male + BMI", data=cases).fit()
    lm_oc90_psqi   = smf.ols("log_OC90 ~ PSQI + Age + Sex_male + BMI", data=cases).fit()

    # Table 4: DHI ~ biomarkers (separate and joint)
    lm_dhi_otolin = smf.ols("DHI ~ log_Otolin1 + Age + Sex_male + BMI", data=cases).fit()
    lm_dhi_oc90   = smf.ols("DHI ~ log_OC90 + Age + Sex_male + BMI", data=cases).fit()
    lm_dhi_joint  = smf.ols("DHI ~ log_Otolin1 + log_OC90 + Age + Sex_male + BMI", data=cases).fit()

    # Collinearity check (VIF) for joint model
    X = lm_dhi_joint.model.exog
    from statsmodels.stats.outliers_influence import variance_inflation_factor
    vif = pd.DataFrame({
        "term": lm_dhi_joint.model.exog_names,
        "VIF": [variance_inflation_factor(X, i) for i in range(X.shape[1])]
    })
    vif.to_csv(os.path.join(OUTDIR, "VIF_joint_model.csv"), index=False)

    # Table 5: nested joint models PSQI + biomarkers for DHI
    mA = smf.ols("DHI ~ PSQI + Age + Sex_male + BMI", data=cases).fit()
```

```
    mB = smf.ols("DHI ~ PSQI + log_Otolin1 + Age + Sex_male + BMI", data=cases).fit()
    mC = smf.ols("DHI ~ PSQI + log_OC90 + Age + Sex_male + BMI", data=cases).fit()
    mD = smf.ols("DHI ~ PSQI + log_Otolin1 + log_OC90 + Age + Sex_male + BMI", data=cases).fit()

    nested = pd.DataFrame([
        {"Model": "A", "PSQI_beta": mA.params["PSQI"], "PSQI_p": mA.pvalues["PSQI"], "R2":
mA.rsquared},
        {"Model": "B", "PSQI_beta": mB.params["PSQI"], "PSQI_p": mB.pvalues["PSQI"], "R2":
mB.rsquared},
        {"Model": "C", "PSQI_beta": mC.params["PSQI"], "PSQI_p": mC.pvalues["PSQI"], "R2":
mC.rsquared},
        {"Model": "D", "PSQI_beta": mD.params["PSQI"], "PSQI_p": mD.pvalues["PSQI"], "R2":
mD.rsquared},
    ])
    nested.to_csv(os.path.join(OUTDIR, "Table5_nested_models.csv"), index=False)

    # -----------------------
    # 7) Exploratory mediation (bootstrap)
    #    Exposure: PSQI; Mediator: log biomarker; Outcome: DHI
    # -----------------------
    def mediation_bootstrap(data_in, mediator_col, n_boot=5000, seed=1):
        rng = np.random.default_rng(seed)
        # Models:
        #   M ~ X + cov
        #   Y ~ X + M + cov
        # Indirect = a*b
        cov_terms = "Age + Sex_male + BMI"
        m_med = smf.ols(f"{mediator_col} ~ PSQI + {cov_terms}", data=data_in).fit()
        m_out = smf.ols(f"DHI ~ PSQI + {mediator_col} + {cov_terms}", data=data_in).fit()
        a = m_med.params["PSQI"]
        b = m_out.params[mediator_col]
        indirect = a * b
        direct = m_out.params["PSQI"]
        total = smf.ols(f"DHI ~ PSQI + {cov_terms}", data=data_in).fit().params["PSQI"]
        prop = indirect / total if total != 0 else np.nan

        boots = []
        for _ in range(n_boot):
            samp = data_in.sample(frac=1.0, replace=True, random_state=int(rng.integers(0,
1_000_000)))
            m_med_b = smf.ols(f"{mediator_col} ~ PSQI + {cov_terms}", data=samp).fit()
            m_out_b = smf.ols(f"DHI ~ PSQI + {mediator_col} + {cov_terms}", data=samp).fit()
            boots.append(m_med_b.params["PSQI"] * m_out_b.params[mediator_col])
        boots = np.array(boots)
        ci = np.quantile(boots, [0.025, 0.975])
        p = (np.mean(boots <= 0) * 2) if indirect > 0 else (np.mean(boots >= 0) * 2)
        return {"mediator": mediator_col, "total": total, "direct": direct, "indirect": indirect,
                "indirect_ci_low": ci[0], "indirect_ci_high": ci[1], "prop_mediated": prop,
"p_boot": p}

    med_otolin = mediation_bootstrap(cases.dropna(subset=["DHI", "PSQI", "log_Otolin1", "Age",
"BMI", "Sex_male"]),
                                     "log_Otolin1", n_boot=5000, seed=10)
    med_oc90   = mediation_bootstrap(cases.dropna(subset=["DHI", "PSQI", "log_OC90", "Age", "BMI",
"Sex_male"]),
                                     "log_OC90", n_boot=5000, seed=11)
    pd.DataFrame([med_otolin, med_oc90]).to_csv(os.path.join(OUTDIR, "Table7_mediation.csv"),
index=False)

# -----------------------
# 8) Sensitivity / subgroup (optional)
# -----------------------
# Exclude recurrent BPPV (if Recurrent column exists: 1 = recurrent)
if "Recurrent" in cases.columns:
    first_episode = cases[cases["Recurrent"] != 1].copy()
    # Repeat key model as needed, e.g., DHI ~ PSQI + covariates
    # (Add additional subgroup analyses similarly)
```

```
print("Pipeline complete. Outputs saved to:", OUTDIR)
```
