# Supplementary material for: Association of sleep quality and inner-ear–specific biomarkers Otolin-1 and otoconin-90 with disease severity in benign paroxysmal positional vertigo
Source: Front Med (Lausanne). 2026 Feb 27;13:1769063. doi: 10.3389/fmed.2026.1769063 (PMC12982452; doi:10.3389/fmed.2026.1769063)
Supplement: Supplementary file 2 [file Data_Sheet_2.docx]

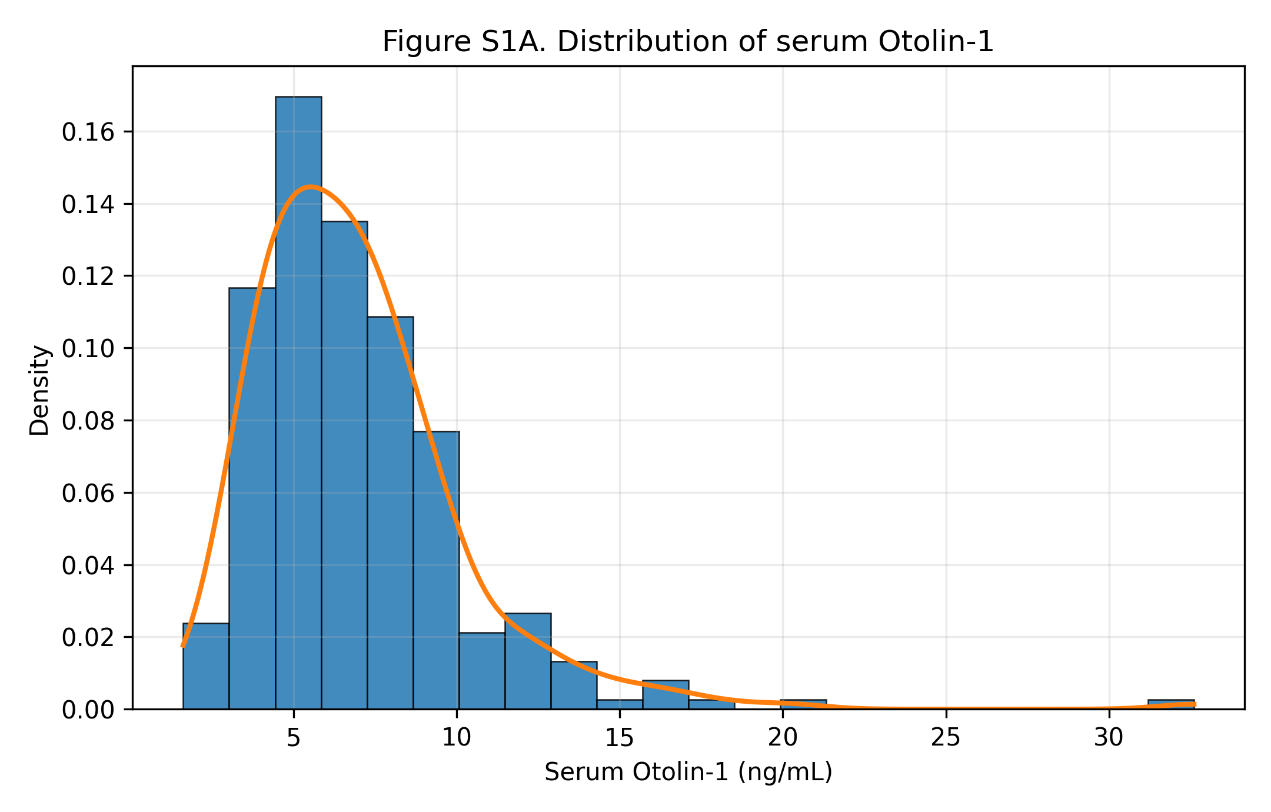


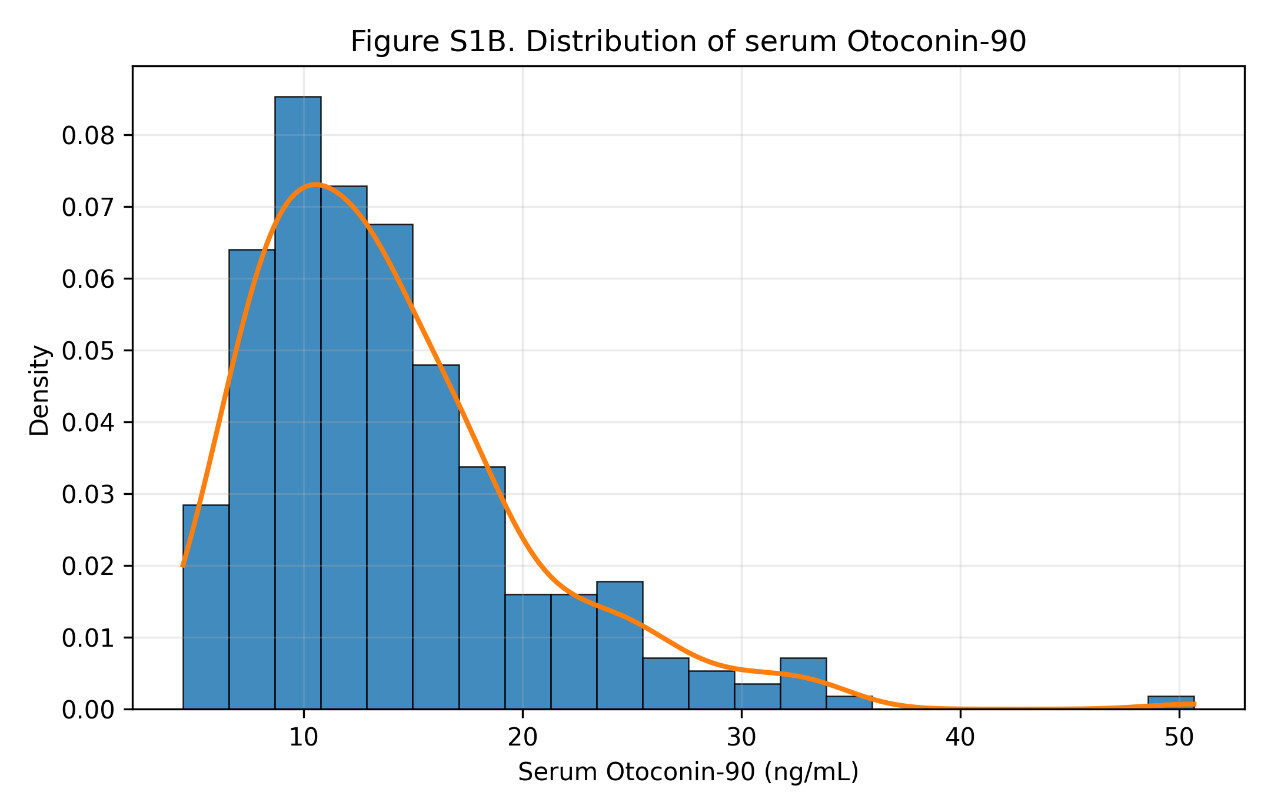


Supplementary **Figure S1. Distribution of serum inner-ear biomarkers.**
**(A)** Histogram and kernel density estimate of serum **Otolin-1** levels.
**(B)** Histogram and kernel density estimate of serum **Otoconin-90 (OC90)** levels.
Both biomarkers exhibited **right-skewed distributions** and were therefore **log-transformed for subsequent analyses**.

Supplementary **Table S1. Exploratory Multiple Linear Regression Analyses of PSQI Components in Relation to Vertigo Severity**

**Outcome variables:** Total DHI score, DHI physical domain (P), DHI emotional domain (E), and DHI functional domain (F)

| **PSQI Component** | **Total DHI β (95% CI)** | **P value** | **DHI-P β (95% CI)** | **P value** | **DHI-E β (95% CI)** | **P value** | **DHI-F β (95% CI)** | **P value** |
| --- | --- | --- | --- | --- | --- | --- | --- | --- |
| Subjective sleep quality | 1.12 (0.38 to 1.86) | 0.003 | 0.42 (0.12 to 0.73) | 0.006 | 0.51 (0.18 to 0.85) | 0.002 | 0.19 (−0.06 to 0.45) | 0.138 |
| Sleep latency | 0.94 (0.21 to 1.67) | 0.012 | 0.35 (0.05 to 0.66) | 0.023 | 0.29 (−0.01 to 0.59) | 0.058 | 0.30 (0.03 to 0.57) | 0.029 |
| Sleep duration | 0.48 (−0.19 to 1.15) | 0.161 | 0.17 (−0.09 to 0.43) | 0.198 | 0.21 (−0.08 to 0.50) | 0.153 | 0.10 (−0.15 to 0.35) | 0.433 |
| Habitual sleep efficiency | 0.76 (0.12 to 1.40) | 0.020 | 0.28 (0.04 to 0.52) | 0.022 | 0.33 (0.06 to 0.61) | 0.018 | 0.15 (−0.08 to 0.38) | 0.204 |
| Sleep disturbances | 1.26 (0.52 to 2.00) | 0.001 | 0.47 (0.17 to 0.78) | 0.002 | 0.55 (0.21 to 0.89) | 0.001 | 0.24 (−0.02 to 0.50) | 0.072 |
| Hypnotic medication use | 0.29 (−0.36 to 0.94) | 0.383 | 0.09 (−0.17 to 0.35) | 0.503 | 0.11 (−0.18 to 0.40) | 0.462 | 0.08 (−0.16 to 0.33) | 0.511 |
| Daytime dysfunction | 2.08 (1.31 to 2.85) | <0.001 | 0.81 (0.49 to 1.12) | <0.001 | 0.69 (0.36 to 1.02) | <0.001 | 0.58 (0.28 to 0.88) | <0.001 |

**Table Notes**

This table presents **exploratory multiple linear regression analyses** examining associations between individual **PSQI component scores** and vertigo severity outcomes.
The **global PSQI score was not included** in these models to avoid collinearity, as it is derived from the component scores.
All models were adjusted for **age, sex, body mass index, disease duration, and affected semicircular canal type**.
Results are expressed as **β coefficients with 95% confidence intervals (CI)**, representing the estimated change in DHI score per 1-point increase in each PSQI component score.
All statistical tests were two-sided, and **P values are reported to three decimal places**.
PSQI indicates **Pittsburgh Sleep Quality Index**; DHI, **Dizziness Handicap Inventory**.

Supplementary **Table S2. Sensitivity Analysis Excluding Patients with Recurrent BPPV**

| **Model** | **Exposure Variable** | **Outcome Variable** | **β (95% Confidence Interval)** | **P value** |
| --- | --- | --- | --- | --- |
| Unadjusted | PSQI score (per 1-point increase) | Total DHI score | 2.26 (1.61 to 2.91) | <0.001 |
| Adjusted† | PSQI score (per 1-point increase) | Total DHI score | 2.01 (1.38 to 2.64) | <0.001 |
| Adjusted† | Serum Otolin-1 (log-transformed) | Total DHI score | 8.12 (4.76 to 11.48) | <0.001 |
| Adjusted† | Serum Otoconin-90 (log-transformed) | Total DHI score | 5.71 (2.54 to 8.88) | <0.001 |

**Table Notes**

This sensitivity analysis was conducted after **excluding patients with recurrent benign paroxysmal positional vertigo (BPPV)**.
Multiple linear regression models were fitted with **Dizziness Handicap Inventory (DHI) total score** as the dependent variable.
All adjusted models were controlled for **age, sex, body mass index, disease duration, and affected semicircular canal type**.
Regression coefficients are presented as **β values with 95% confidence intervals (CI)**.
All tests were two-sided, with **P values reported to three decimal places**.
PSQI indicates **Pittsburgh Sleep Quality Index**; DHI, **Dizziness Handicap Inventory**.

Supplementary **Table S3. Subgroup Analyses Stratified by Semicircular Canal Type and Sex**

**A. Stratified by Affected Semicircular Canal Type**

| **Subgroup** | **Exposure Variable** | **β (95% Confidence Interval)** | **P value** |
| --- | --- | --- | --- |
| Posterior canal | PSQI score | 2.18 (1.46 to 2.90) | <0.001 |
|  | Otolin-1 (log-transformed) | 8.47 (4.91 to 12.03) | <0.001 |
|  | Otoconin-90 (log-transformed) | 6.08 (2.72 to 9.44) | <0.001 |
| Non-posterior canal | PSQI score | 1.94 (1.03 to 2.85) | <0.001 |
|  | Otolin-1 (log-transformed) | 7.61 (3.28 to 11.94) | 0.001 |
|  | Otoconin-90 (log-transformed) | 5.22 (1.48 to 8.96) | 0.007 |

**B. Stratified by Sex**

| **Subgroup** | **Exposure Variable** | **β (95% Confidence Interval)** | **P value** |
| --- | --- | --- | --- |
| Male | PSQI score | 2.05 (1.18 to 2.92) | <0.001 |
|  | Otolin-1 (log-transformed) | 8.29 (4.12 to 12.46) | <0.001 |
|  | Otoconin-90 (log-transformed) | 5.87 (2.09 to 9.65) | 0.002 |
| Female | PSQI score | 2.11 (1.34 to 2.88) | <0.001 |
|  | Otolin-1 (log-transformed) | 8.01 (4.37 to 11.65) | <0.001 |
|  | Otoconin-90 (log-transformed) | 5.63 (2.31 to 8.95) | 0.001 |

**Table Notes**

Subgroup analyses were performed by **affected semicircular canal type** and **sex** to assess the robustness of the associations between sleep quality, inner-ear structural biomarkers, and vertigo severity.
All models were adjusted for **age, body mass index, disease duration**, and other relevant clinical covariates where appropriate.
Results are expressed as **β coefficients with 95% confidence intervals (CI)**.
No statistically significant interaction effects were observed between subgroup variables and the main exposures.
All tests were two-sided, with **P values reported to three decimal places**.
